# Frailty Assessment in Polymyalgia Rheumatica: Comparative Performance and Determinants of Clinical-Research Frailty Assessment (CRAF) and Kihon Checklist (KCL) Scores

**DOI:** 10.3390/jcm15166403

**Published:** 2026-08-19

**Authors:** Fausto Salaffi, Marina Carotti, Sonia Farah, Francesca Bandinelli

**Affiliations:** 1 Rheumatology Unit, Department of Clinical and Molecular Sciences, Carlo Urbani Hospital, Università Politecnica of Marche, 60035 Jesi, Italy; fausto.salaffi@gmail.com (F.S.);; 2 Radiology Department, Ospedali Riuniti, Università Politecnica Delle Marche, 60126 Ancona, Italy; 3 Rheumatology Division, Department of Rheumatology, Santa Maria Nuova Hospital, Local Health Unit (USL) Tuscany Center, 50122 Florence, Italy

**Keywords:** polymyalgia rheumatica, frailty, comorbidity, disease activity

## Abstract

**Background/Objectives:** Frailty is increasingly recognized as an important condition in polymyalgia rheumatica (PMR), although different instruments may assess different dimensions of vulnerability. This study aimed to compare the determinants and clinical correlates of frailty measured by the Comprehensive Rheumatologic Assessment of Frailty (CRAF) and the Italian Kihon Checklist (I-KCL). **Methods:** Fifty-two patients with PMR were evaluated. The discriminative performance of CRAF and I-KCL was assessed against the Cardiovascular Health Study (CHS) frailty phenotype using ROC analysis. Multivariable linear regression identified the independent determinants of each frailty measure. **Results:** The distribution of frailty status varied across the three assessment tools. The CRAF score identified a higher proportion of non-frail patients (44.2%) and a lower proportion of frail individuals (19.2%) compared to both the I-KLC and CHS criteria. Discriminative accuracy of CRAF and I-KCL scores against the CHS/Fried phenotype reference standard was excellent for both instruments: CRAF AUC = 0.96 (95% CI 0.888–1, bootstrap, 2000 stratified resamples) and I-KCL AUC = 0.899 (95% CI 0.704–1). The difference between CRAF and I-KCL discriminative accuracy was therefore not statistically significant (z = 0.941, *p* = 0.3467). In multivariable linear regression models adjusted for age, disease duration, PMR-AS(CRP), comorbidity count, HAQ, and fatigue, PMR-AS(CRP) (standardized β = 0.51, *p* < 0.001) and comorbidity count (standardized β = 0.36, *p* < 0.001) were independently associated with the CRAF score (adjusted R^2^ = 0.787, n = 51), while PMR-AS(CRP) (standardized β = 0.40, *p* < 0.001) and comorbidity count (standardized β = 0.46, *p* < 0.001) were independently associated with the I-KCL score (adjusted R^2^ = 0.675, n = 51). A further finding was that functional impairment, measured by the HAQ, was independently associated with the CRAF score (standardized β = 0.19, *p* = 0.018) but not with the I-KCL score (standardized β = 0.06, *p* = 0.512). **Conclusions:** Although CRAF and I-KCL share these core determinants, they capture different dimensions of frailty: CRAF is more strongly influenced by functional impairment, whereas I-KCL appears less affected by disability. These instruments should therefore be considered complementary rather than interchangeable.

## 1. Introduction

PMR is a common inflammatory rheumatic disease of older adults, characterized by bilateral shoulder and pelvic girdle pain and stiffness, elevated acute-phase reactants, and a typically prompt response to low-dose glucocorticoids [1,2]. The disease predominantly affects individuals over 50 years of age, with incidence rising steeply with advancing age, and it is frequently complicated by relapse, prolonged glucocorticoid exposure, and glucocorticoid-related morbidity [2,3].

Classification is currently based on the 2012 EULAR/ACR provisional criteria [1], while musculoskeletal ultrasound has emerged as a valuable adjunct for confirming bursitis and tenosynovitis and for distinguishing PMR from mimicking conditions such as late-onset rheumatoid arthritis, remitting seronegative symmetrical synovitis with pitting oedema (RS3PE), and other inflammatory or degenerative disorders of the shoulder and hip girdles [3,4].

Frailty is a state of increased vulnerability to physiological stressors, resulting from cumulative decline across multiple organ systems, and is associated with a heightened risk of disability, hospitalization, and mortality [5]. Two complementary conceptual models dominate the field: the physical phenotype model of Fried et al., which operationalizes frailty as a categorical syndrome defined by unintentional weight loss, exhaustion, weakness, slowness, and low physical activity [6], and the deficit-accumulation model of Rockwood and Mitnitski, which quantifies frailty as a continuous index reflecting the proportion of accumulated health deficits across a broad multidimensional inventory [7]. Chronic low-grade systemic inflammation, or “inflammaging,” has been proposed as a key biological substrate linking ageing and frailty, with elevated circulating interleukin-6 (IL-6), tumour necrosis factor-α (TNF-α), and C-Reactive Protein consistently associated with both frailty phenotype and frailty index status in community-dwelling older adults [8,9,10]. Because PMR is, by definition, a systemic inflammatory disease characterized by persistently elevated IL-6 and CRP, patients with PMR are biologically plausible candidates for accelerated frailty trajectories, independent of chronological age [8,9]. Consistent with this hypothesis, frailty has been shown to be highly prevalent among patients with PMR: in a cohort study using the Fried phenotype, Sattui et al. reported frailty or pre-frailty in the majority of patients with PMR, with frailty status independently associated with worse health-related quality of life, cognitive performance, and sarcopenia [11]. Similar evidence of excess frailty burden has accumulated across other immune-mediated rheumatic diseases, including rheumatoid arthritis, where frailty is associated with greater disease activity, disability, and adverse outcomes [12], prompting recent calls for frailty assessment to be systematically incorporated into rheumatology clinical practice and research [13]. Despite this growing recognition, no dedicated, rheumatology-specific frailty instrument had been validated in PMR.

A substantial number of frailty assessment tools are currently available, including the Fried physical phenotype [6]. The Rockwood Frailty Index [14], the Edmonton Frail Scale [15], the Survey of Health, Ageing and Retirement in Europe-Frailty Instrument (SHARE-FI) [16] and the Tilburg Frailty Indicator [17], among others. These instruments differ substantially in their theoretical underpinnings, item content, administration burden, and intended care setting, and none was developed or validated specifically in populations with inflammatory rheumatic disease, where disease-related pain, fatigue, and disability can overlap with and confound generic frailty domains. The Fried/Cardiovascular Health Study (CHS) phenotype was selected as the reference standard for the present study because it provides an operationally explicit, dichotomous (frail/non-frail) classification with well-established construct and predictive validity across multiple populations [5,6], a property that is methodologically necessary for the receiver operating characteristic (ROC)-based discriminative analyses that are central to this study; continuous, weighted indices such as the Rockwood Frailty Index, while conceptually appealing, do not yield a single universally accepted dichotomization threshold and were therefore considered less suitable as an external criterion in this context [7]. To address the absence of a rheumatology-specific instrument, our group developed and validated the CRAF, a multidimensional frailty screening tool incorporating disease-related items alongside classical frailty domains, in a cohort of patients with rheumatoid arthritis [18], where advanced age and higher disease activity were independently associated with frailty status. Whether the CRAF performs adequately outside the disease setting in which it was originally validated—and specifically in PMR, a disease with a distinct demographic, inflammatory, and glucocorticoid-exposure profile—has not previously been examined.

The Kihon Checklist is a 25-item self-administered questionnaire developed in Japan for population-level frailty and long-term-care-risk screening, covering physical, nutritional, cognitive, and mood-related domains; it has demonstrated good validity against the Fried phenotype and other frailty measures in community-dwelling older adults [19], and an Italian version I-KCL [20] is increasingly used in geriatric and primary-care screening in Italy. However, as with the CRAF, the discriminative and construct validity of the I-KCL had not previously been evaluated in a PMR population.

Taken together, these considerations define three specific and, to our knowledge, previously unaddressed gaps in the literature: first, the degree of categorical and score-level agreement between the CRAF and the I-KCL has not been established in any patient population; second, the comparative discriminative performance of the two instruments against an accepted external reference standard of frailty has not been directly tested, in PMR or elsewhere; and third, the pattern of association between each instrument’s score and clinically relevant PMR-specific variables (disease activity, disease duration, comorbidity burden, functional disability, and fatigue) has not been characterized, precluding an evidence-based recommendation on which tool—if either—is preferable for frailty screening in this population. This is also, to our knowledge, the first study to evaluate the CRAF outside the rheumatoid arthritis population in which it was originally developed [18], and the first head-to-head comparison of the CRAF and the I-KCL in any clinical setting. Accordingly, the present study aimed to compare the CRAF and the I-KCL in a cohort of patients with PMR with respect to (i) categorical and continuous agreement, (ii) discriminative accuracy against the Fried/CHS phenotype as reference standard, and (iii) construct validity against demographic, clinical, and disease-activity variables, in order to inform the choice of frailty screening instrument for use in PMR clinical practice and research.

## 2. Materials and Methods

### 2.1. Study Design and Participants

The study enrolled 52 patients aged 50 to 80 years, diagnosed with PMR according to the 2012 EULAR/ACR Provisional Classification Criteria (1,2). Exclusion criteria included cognitive impairments, a lack of command of the Italian language, and concurrent disorders such as severe chronic obstructive pulmonary disease, heart disease, multiple sclerosis, extracorporeal dialysis, fibromyalgia, or chronic infectious illnesses. All patients were receiving oral corticosteroids, with a mean prednisone (or equivalent) dose of 5.7 mg/day (range: 2.5–20). Patients, initially sent by general practitioners, were included after privacy and informed consent (saved in the electronic chart for anonymous analysis and publication of routine clinical data). All procedures performed in this study complied with standards of the Italian institutional research ethical rules, Italian Health Ministry legislation (law of 22 April 2021, no. 52, valid until repeal of law of 10 August 2023, no. 105) and the Declaration of Helsinki.

### 2.2. Measurements

In patients with PMR, frailty was initially assessed in all cases according to the CHS criteria for frailty status [6]. All patients subsequently completed the CRAF [18] and the I-KCL frailty index [20] following the initial evaluation.

*CHS Criteria for Frailty Status*: frailty status was defined according to the CHS/Fried physical frailty phenotype, based on five dimensions—shrinking, exhaustion, low physical activity, weakness, and slowness—as originally described by Fried et al. [6]. Participants meeting none of the five criteria were classified as robust/non-frail (score 0), those meeting one or two criteria as pre-frail (score 1–2), and those meeting three or more criteria as frail (score 3–5). Shrinking was defined as self-reported unintentional weight loss of more than 4.5 kg during the preceding year. Exhaustion was assessed using a single question—“Have you felt tired or fatigued without reason during the past two weeks?”—with a positive response taken to indicate low energy/exhaustion. This single-item assessment is a pragmatic, non-standard substitute for the original CHS exhaustion criterion, which is operationalized using two items from the Center for Epidemiologic Studies Depression Scale (CES-D) [21], asking how often in the past week the respondent felt that “everything I did was an effort” or “I could not get going,” with exhaustion defined as endorsing either item “a moderate amount of the time” or “most of the time.” Our single-item substitute has not itself been formally validated against this two-item definition in PMR or in other populations, and may have led to misclassification of the exhaustion criterion; this is acknowledged as a limitation.

*Physical activity*: low physical activity was assessed using the Italian version of the Health Assessment Questionnaire (HAQ) [22,23,24], used here as a pragmatic proxy for the Minnesota Leisure Time Activity Questionnaire originally employed in the CHS [23], because a validated, activity-specific instrument corresponding to the Minnesota questionnaire was not available in our setting. We note that low physical activity, in the CHS framework, is operationally difficult to disentangle from disability-driven activity limitation in patients with active inflammatory joint/muscle disease, and HAQ scores have previously been shown to correlate with objective and self-reported physical activity levels in inflammatory rheumatic disease [22,23,24]. However, because the HAQ primarily captures disability in activities of daily living rather than voluntary leisure-time energy expenditure, this substitution may partly conflate two conceptually distinct CHS criteria—physical disability and low activity—through a shared measurement instrument; this construct overlap is discussed further as a limitation, together with its implications for the interpretation of associations between frailty status and functional outcomes. The HAQ assesses the degree of difficulty a person has in accomplishing tasks in eight functional domains: dressing and grooming, arising, eating, walking, hygiene, reach, grip, and other activities. For each domain, participants rate their level of difficulty over the preceding week on a 4-point scale ranging from 0 (no difficulty) to 3 (unable to perform). The eight domain scores are summed and divided by eight to yield the HAQ Disability Index (HAQ-DI), ranging from 0 to 3, with higher scores indicating greater disability [24].

*Measurement of handgrip strength (HGS)*: HGS was assessed using a custom-built cylindrical grip dynamometer equipped with five force-sensitive resistors (FSR-402, Interlink Electronics, Camarillo, CA, USA) connected to an Arduino Mega 2560 microcontroller. Unlike conventional dynamometers that provide only an instantaneous peak force, this device continuously records grip force over a 30 s period, allowing the assessment of sustained grip performance through repeated measurements from each sensor during the acquisition period. HGS was measured twice in the dominant hand, and participants were allowed a 5 min rest interval between trials to minimize the effects of muscle fatigue. The highest value obtained from the two attempts was used for the statistical analyses, consistent with current recommendations for HGS assessment. All measurements were performed according to the standardized protocol of the American Society of Hand Therapists (ASHT) [25], with participants seated, the shoulder adducted and neutrally rotated, the elbow flexed at approximately 90°, the forearm in a neutral position, and the wrist maintained in slight extension (0–30°) with 0–15° of ulnar deviation. Standardized verbal instructions and encouragement were provided to all participants to ensure uniform testing conditions [26,27]. For the present study, weak HGS was defined as values within the lowest sex-specific quartile of the study population. The corresponding cut-off values were <33.3 kg for men and <19.8 kg for women. Although these thresholds were derived empirically from the study cohort, HGS is widely recognized as a reliable indicator of overall muscle strength and physical function and represents a core component of contemporary frailty and sarcopenia assessment [28,29,30].

*CRAF*: the CRAF includes ten variables: the score ranges between 0.0 (no deficit present) and 1.0 (deficit present) [18]. Some variables (nutritional status, polypharmacy, and social activity) were considered as trichotomous levels (0, 0.5, 1); while other variables, such as comorbidity and those related to the patient’s perspective (pain, fatigue, physical function, and depression) were assigned five (0, 0.25, 0.5, 0.75 and 1) to six levels (0, 0.2, 0.4, 0.6, 0.8 and 1), to reflect differences in severity. Regarding the nutritional status, overweight and normal weight were combined and coded 0, based on evidence that some excess weight can be protective. Obesity was coded 0.5, while underweight was 1.0. The ten scores were added and divided by the total number of deficits evaluated to produce a CRAF index between 0.0 (i.e., no deficits present) and 1.0 (i.e., all deficits present). The calculation of the CRAF is based on the sum of the ten variables divided by ten; the score ranges between 0.0 (no deficit present) and 1.0 (all deficits present). The CRAF cut-off points have been established using Clegg’s criteria [31], as follows: score ≤ 0.12 represents patients without frailty; score > 0.12 and ≤0.24 represents patients with mild frailty; score > 0.24 and ≤0.36 represents patients with moderate frailty; and score > 0.36 represents patients with severe frailty. For this study, we have grouped the mild and moderate frailty categories into a single category of patients with pre-frailty.

*I-KCL*: the I-KCL (Table S1, in Supplementary Materials) consists of 25 items (yes/no) divided into seven categories: physical strength, nutrition, eating, socialization, memory, mood and lifestyle (questions 1–20). Each category is rated on a pass (0)/fail (1) basis, and the sum of all indices ranges from 0 (no frailty) to 25 (severe frailty) (Table 1); a higher score indicates worse functioning [32,33,34]. The time required for older adults to answer the KCL is approximately 15 min. In patients with rheumatoid arthritis, a cut-off point ≥7 on the KCL gave the best trade-off for sensitivity (93.3; CI 95% 81.7 to 98.6) and specificity (90.8; CI 95% 85.5 to 94.7); LR+ 10.15, LR− 0.073 and the AUC was 0.928 (SE 0.0216, *p* < 0.001, CI 95% 0.886 to 0.970) [20].

### 2.3. Statistical Analysis

For sample-size calculation, we report a post hoc power calculation based on the AUCs actually observed for CRAF and I-KCL against the CHS/Fried physical frailty phenotype reference standard [6], using the closed-form Hanley and McNeil standard-error formula [35]. With 52 patients enrolled (9 frail, 43 non-frail by the CHS phenotype; frailty prevalence 17.3%), the achieved sample provided >99% power to detect the observed CRAF AUC of 0.960 and >99% power to detect the observed I-KCL AUC of 0.899, each against the null value of 0.5, at a two-sided α of 0.05. Applying the same method, a minimum of 10 patients (2 frail, 8 non-frail) would have been required to detect the CRAF AUC, and 15 patients (3 frail, 12 non-frail) to detect the I-KCL AUC, with 80% power at this prevalence—both well below the 52 patients actually enrolled. This is a post hoc power analysis, appropriate for characterizing the precision achieved by the completed study, using a reference comparable sample size (41 PMR patients included) of Sattui et al. [11]; it should not be read as a substitute for prospective sample-size planning in future confirmatory studies, for which more conservative effect-size assumptions are recommended. The normality of continuous variables was assessed using the Shapiro–Wilk test [36], which is preferred over the Kolmogorov–Smirnov test for a sample of this size (n = 52), the latter having low power and requiring a Lilliefors correction in small samples [37]. Descriptive statistics were presented as mean and standard deviation, or median with 95% confidence interval (95% CI), depending on the distribution of the data, for continuous parameters, and as frequencies and percentages for categorical variables, as appropriate. Histograms were used to visualize score distributions. Demographic and clinical measures were compared between groups using the Mann–Whitney U test or the Kruskal–Wallis test for continuous variables, as appropriate to the number of groups and the distribution of the data. Categorical data were compared using the χ^2^ test, with Fisher’s exact test substituted where expected cell counts were below. The reference standard for frailty was the CHS/Fried physical frailty phenotype. The prevalence of frailty by CRAF was calculated using the categorical cut-offs defined in the original validation study of the instrument (≤0.12, non-frail; 0.12–0.24, mild frailty; 0.24–0.36, moderate frailty; >0.36, severe frailty) [18], with the mild and moderate categories collapsed into a single “pre-frail” category for the purposes of the present cross-tool comparison (see Limitations Section). The prevalence of frailty by I-KCL was calculated using the validated three-category cut-offs of Satake et al. (0–3, robust/non-frail; 4–7, pre-frail; ≥8, frail; maximum score 25) [19]. Receiver operating characteristic (ROC) curve analysis and the corresponding area under the curve (AUC) were used to evaluate the discriminative accuracy of CRAF and I-KCL against the CHS/Fried phenotype. AUC values were interpreted according to Swets: 0.50–0.70, poor accuracy; >0.70–0.90, useful for some purposes; and >0.90, high accuracy. The optimal cut-point for each instrument was defined as the value maximizing the Youden index (sensitivity + specificity − 1). Ninety-five percent confidence intervals for the AUC were estimated using a stratified non-parametric bootstrap with 2000 resamples [38], drawn separately from the frail and non-frail groups to preserve the observed class prevalence in each resample; the 2.5th and 97.5th percentiles of the resulting bootstrap distribution were used as the confidence limits (percentile method). Bootstrap resampling was chosen over closed-form variance estimators because it does not rely on asymptotic normality assumptions that can be unreliable in a relatively small sample (n = 52), and 2000 replicates exceed the minimum of 1000–2000 generally recommended for stable percentile-based confidence-interval estimation. Because the CRAF- and I-KCL-derived ROC curves were generated from the same participants against the same reference standard, the two AUCs are statistically correlated. They were therefore compared using DeLong’s test for correlated ROC curves [39], which accounts for this within-subject correlation through a structural-components covariance estimator, rather than the original Hanley and McNeil approach, which assumes independent, unpaired samples and is not appropriate for paired within-subject ROC comparisons. Multivariable linear regression models were fitted separately for the CRAF and I-KCL scores as continuous dependent variables. Candidate predictors—age, disease duration, PMR-AS(CRP), comorbidity count, HAQ, and fatigue—were selected a priori on clinical grounds and entered simultaneously using a fixed “enter” method, rather than a stepwise or other data-driven selection procedure, to limit the additional overfitting risk associated with automated variable selection in a sample of this size. Both unstandardized (B, SE, 95% CI) and standardized (β, derived from z-scored predictors and outcome) regression coefficients are reported, given the different raw scoring ranges of CRAF (0–1) and I-KCL (0–25), which make direct comparison of unstandardized coefficients between models inappropriate. Multicollinearity was assessed using the variance inflation factor (VIF) for each predictor, with VIF < 5 (and, more conservatively, <2.5) taken to indicate no problematic multicollinearity [40]. Residual diagnostics included the Shapiro–Wilk test for normality of residuals and visual inspection of quantile–quantile (Q-Q) plots. Given the available sample (n = 51 after exclusion of one case with missing HAQ) and six candidate predictors, the resulting subjects-per-variable ratio (approximately 8.5) was below the conventionally recommended threshold of 10–15 observations per predictor; regression estimates are therefore reported as hypothesis-generating, and this limitation is discussed explicitly in the discussion. Current prednisone-equivalent glucocorticoid dose, disease duration, and comorbidity count were considered as potential confounders of the association between disease activity and frailty. Disease duration and comorbidity count were included as covariates in the primary regression models described above; glucocorticoid dose was not entered into the primary models reported here and is addressed as a limitation in the Discussion Section. A dedicated, validated physical-activity instrument (e.g., the Minnesota Leisure Time Activity Questionnaire) and a formal nutritional assessment were not available in the present dataset; the absence of these covariates is likewise acknowledged as a limitation. Data were entered into a Microsoft Access database originally developed for a prospective multicenter study of PMR. The computed statistics were performed in Python, version 3.13.5 using the packages: pandas (version 2.2.3)—data handling and cleaning; scipy.stats (version 1.17.0)—Shapiro–Wilk, Wilcoxon, chi-square, and Spearman; statsmodels (version 0.14.6)—OLS regression and VIF (variance_inflation_factor); scikit-learn (version 1.8.0)—ROC curves and AUC, a custom implementation of DeLong’s test (fastDeLong algorithm), since scikit-learn does not include it natively.

## 3. Results

### 3.1. Characterization of the Patient Population and Score Distributions of the Frailty Indices

Of the 55 PMR individuals initially enrolled in the study, 3 (5.5%) were excluded due to incomplete data, leaving a total of 52 participants for analysis. The final cohort had a mean age of 69.4 ± 8.7 years (range 54–84) and a mean disease duration of 5.6 ± 3.3 months. The majority were female (34 women, 65.4%; 18 men, 34.6%). Comorbidity burden was low to moderate, with a mean of 2.0 ± 2.0 comorbidities per patient (median 1, range 0–7). At baseline, participants showed active disease with a mean of Polymyalgia Rheumatica Activity Score calculated with C-Reactive Protein (PMR-AS—CRP) of 28.0 ± 5.5 and a mean of Polymyalgia Rheumatica Activity Score calculated with Erythrocyte Sedimentation Rate (PMR-AS—ESR) of 28.8 ± 5.9 (41). Inflammatory markers were elevated: CRP 5.9 ± 1.4 mg/dL and ESR 62.7 ± 13.6 mm/h. Patient-reported pain (VAS) was 6.5 ± 1.4 and fatigue 6.2 ± 2.5 on a 0–10 scale. Morning stiffness duration averaged 70.2 ± 23.2 min, and elevation of upper limbs was limited (EUL 2.5 ± 0.6 out of 3). Functional disability assessed by the HAQ was 0.87 ± 0.54 (available in 51/52 patients). At baseline, 27 patients (51.9%) reported an acceptable symptom state (PASS = 1). Frailty status is reported according to the CRAF, I-KCL, and CHS criteria. At baseline, the mean CRAF score was 0.255 (95% confidence interval [CI]: 0.228–0.281), with a median of 0.200 (95% CI: 0.180–0.225). The mean I-KCL score was 5.96 (95% CI: 5.43–6.50), with a median of 5.00 (95% CI: 4.00–5.00).

### 3.2. Prevalence of Frailty

**Figure 1:**
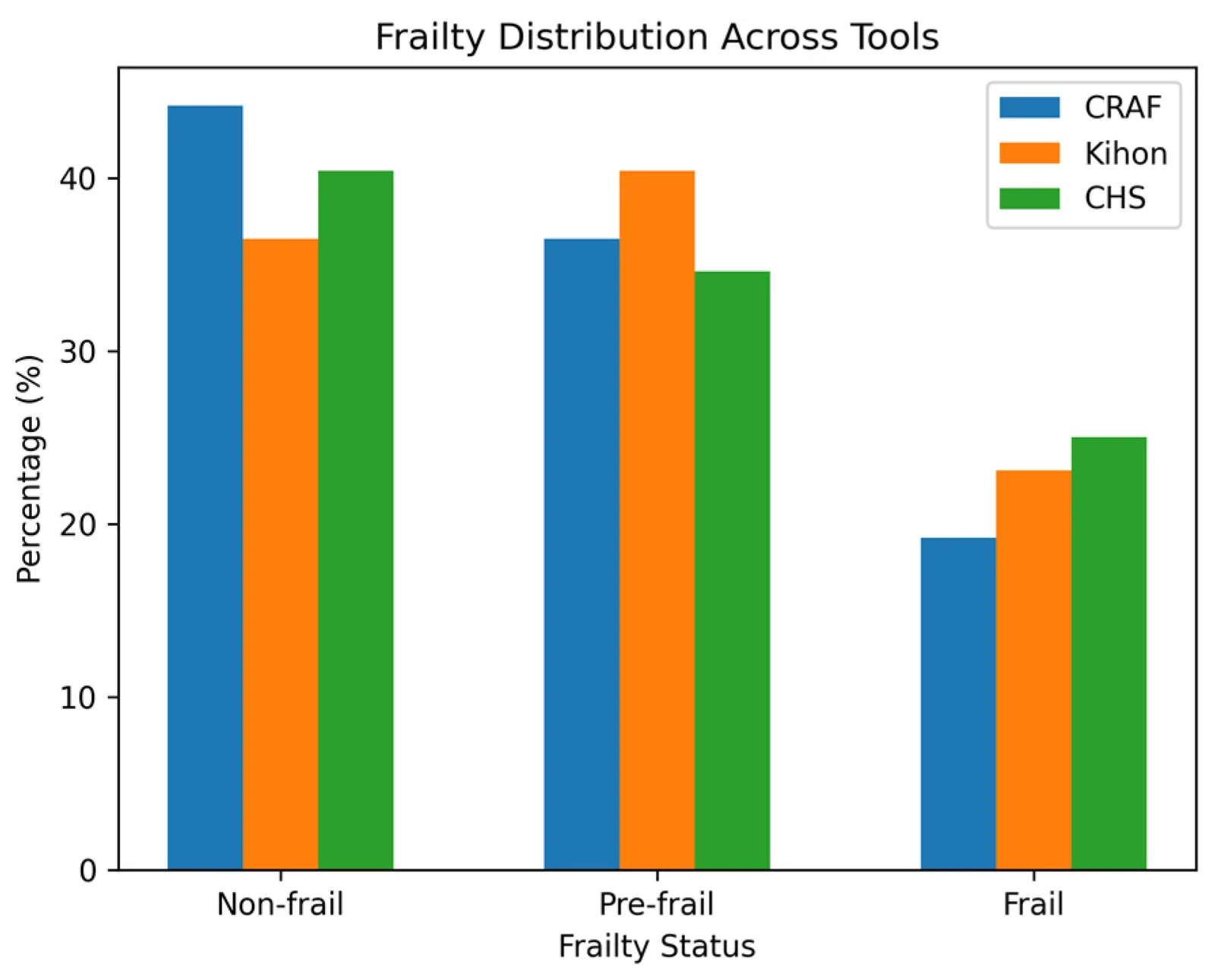
Distribution of frailty status according to CRAF, Kihon Checklist, and CHS criteria. CRAF identified a higher proportion of non-frail patients, whereas CHS classified more patients as frail. The Kihon Checklist showed the highest proportion of pre-frail individuals, highlighting differences in frailty stratification across tools.

The distribution of frailty status varied across the three assessment tools (Figure 1). The CRAF score identified a higher proportion of non-frail patients (44.2%) and a lower proportion of frail individuals (19.2%) compared to both the I-KLC and CHS criteria. In contrast, CHS classified the highest percentage of patients as frail (25.0%), while the I-KLC showed the highest proportion of pre-frail individuals (40.4%). These differences suggest that CRAF may be more conservative in identifying frailty, potentially capturing earlier or milder stages of vulnerability, whereas CHS appears to classify a greater proportion of patients as overtly frail. Overall, the three tools show partial overlap but also highlight the heterogeneity of frailty assessment in patients with PMR (Table 1 and Figure 1). Because CRAF and I-KCL classifications were obtained from the same patients (paired, within-subject design) using a 3-level ordinal outcome (non-frail/pre-frail/frail), Bowker’s test of symmetry—the generalization of McNemar’s test to tables larger than 2 × 2—was used to test whether the two instruments classified patients into the same categories, rather than the standard 2 × 2 McNemar’s test. The 12 discordant patients (6 CRAF non-frail/I-KCL pre-frail, and 2 CRAF pre-frail/I-KCL non-frail) shifted by only one adjacent category in every case, with zero major (non-frail vs. frail) discordances, consistent with the high weighted kappa.

*Multivariable linear regression models*. Two multivariable linear regression models were fitted with CRAF score and I-KCL score, respectively, as the dependent variable. Candidate predictors (age, disease duration, PMR-AS [CRP-based, as a measure of disease activity], number of comorbidities, HAQ score [functional status], and fatigue VAS) were entered simultaneously (“enter” method) based on clinical plausibility and on the variables reported in the Discussion Section of between-tool differences; pain, CRP, and morning stiffness were not entered separately because they are algebraic components of the PMR-AS composite score and their inclusion alongside PMR-AS would have produced built-in circularity/collinearity. One patient with missing HAQ was excluded listwise (n = 51).

**Figure 2:**
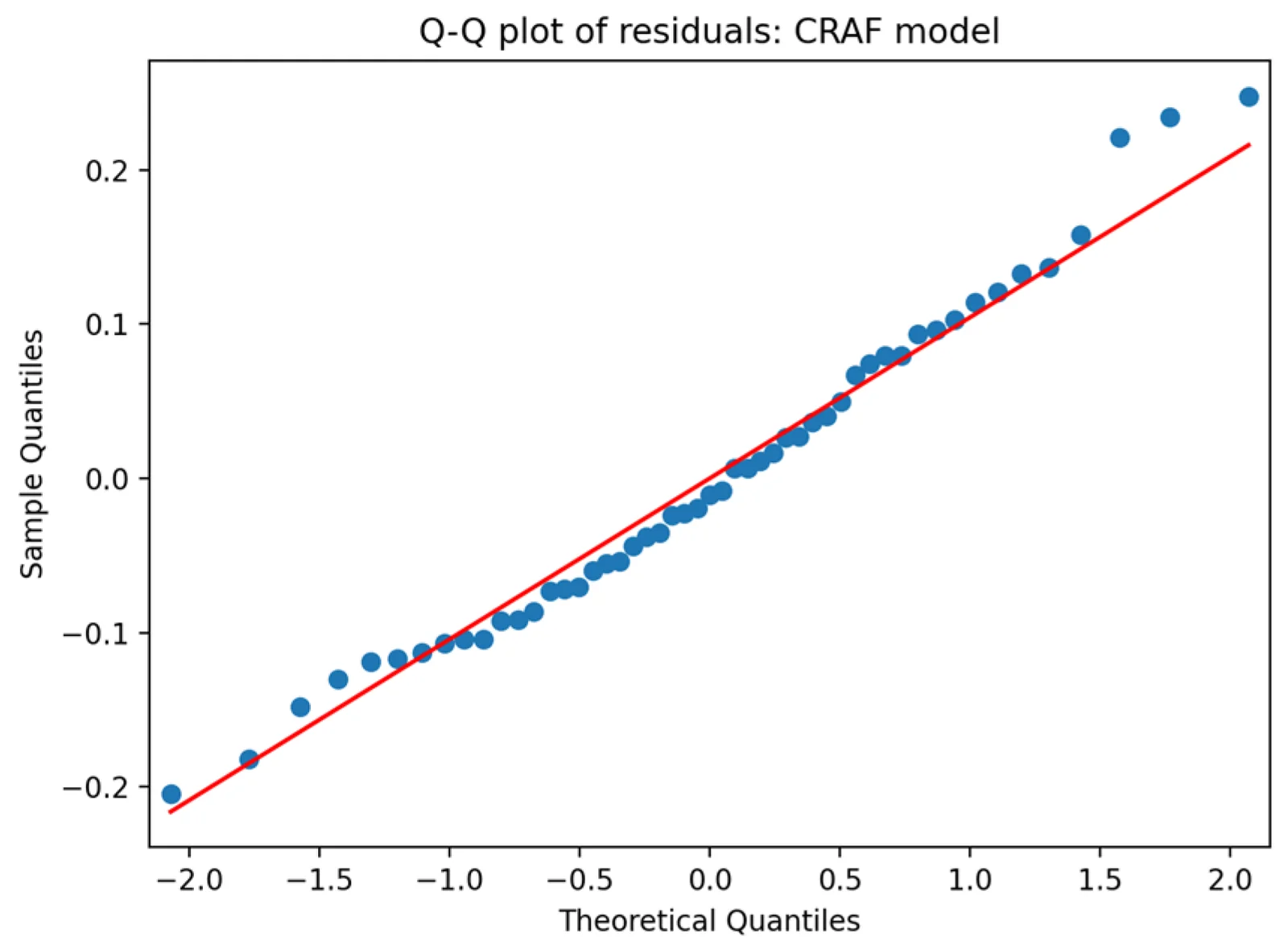
Q-Q plots of standardized residuals for the CRAF regression models.

*CRAF model*: R^2^ = 0.812, adjusted R^2^ = 0.787, F (6,44) = 31.74, *p* < 0.001. Maximum VIF = 2.04 (no problematic multicollinearity, all VIF < 2.1, well below the conventional threshold of 5). Shapiro–Wilk test on model residuals: W = 0.977, *p* = 0.426 (residuals not significantly different from normal; Q-Q plot in Figure 1 shows no material departure from normality). PMR-AS, number of comorbidities, and HAQ were independent, statistically significant predictors of CRAF score; age, disease duration, and fatigue were not (Table 2a and Figure 2).

**Figure 3:**
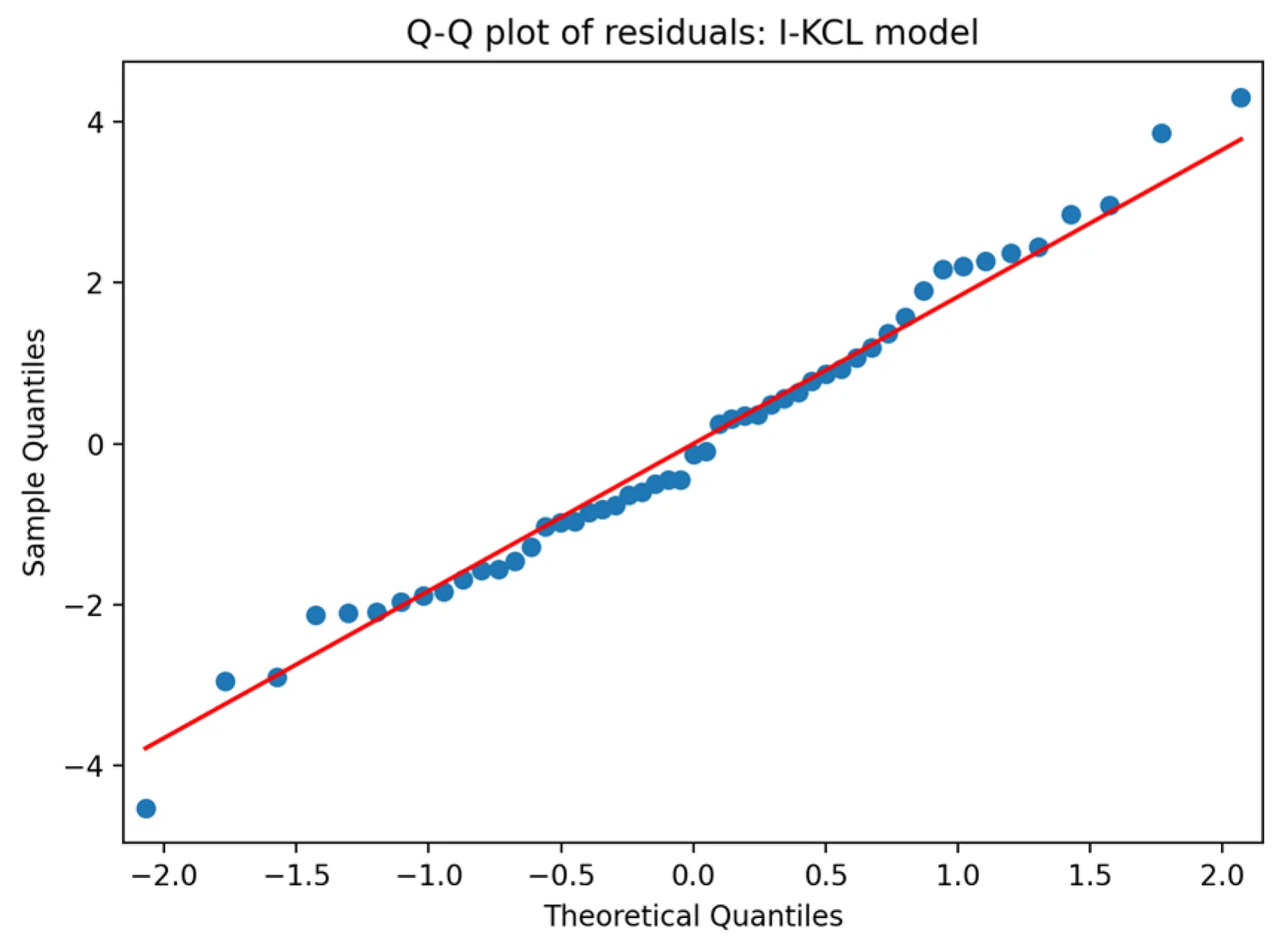
Q-Q plots of standardized residuals for the I-KCL regression models.

*I-KCL model*: R^2^ = 0.714, adjusted R^2^ = 0.675, F (6,44) = 18.34, *p* < 0.001. Maximum VIF = 2.04 (no problematic multicollinearity). Shapiro–Wilk test on residuals: W = 0.988, *p* = 0.882 (residuals approximately normal; Figure 3). As with CRAF, PMR-AS and number of comorbidities were independent significant predictors of I-KCL score, whereas age, disease duration, HAQ, and fatigue were not—notably, HAQ was independently associated with CRAF but not with I-KCL, consistent with HAQ having been used to operationalize the “low physical activity” CHS criterion embedded within CRAF (see Limitations Section) (Table 2b and Figure 3).

**Figure 4:**
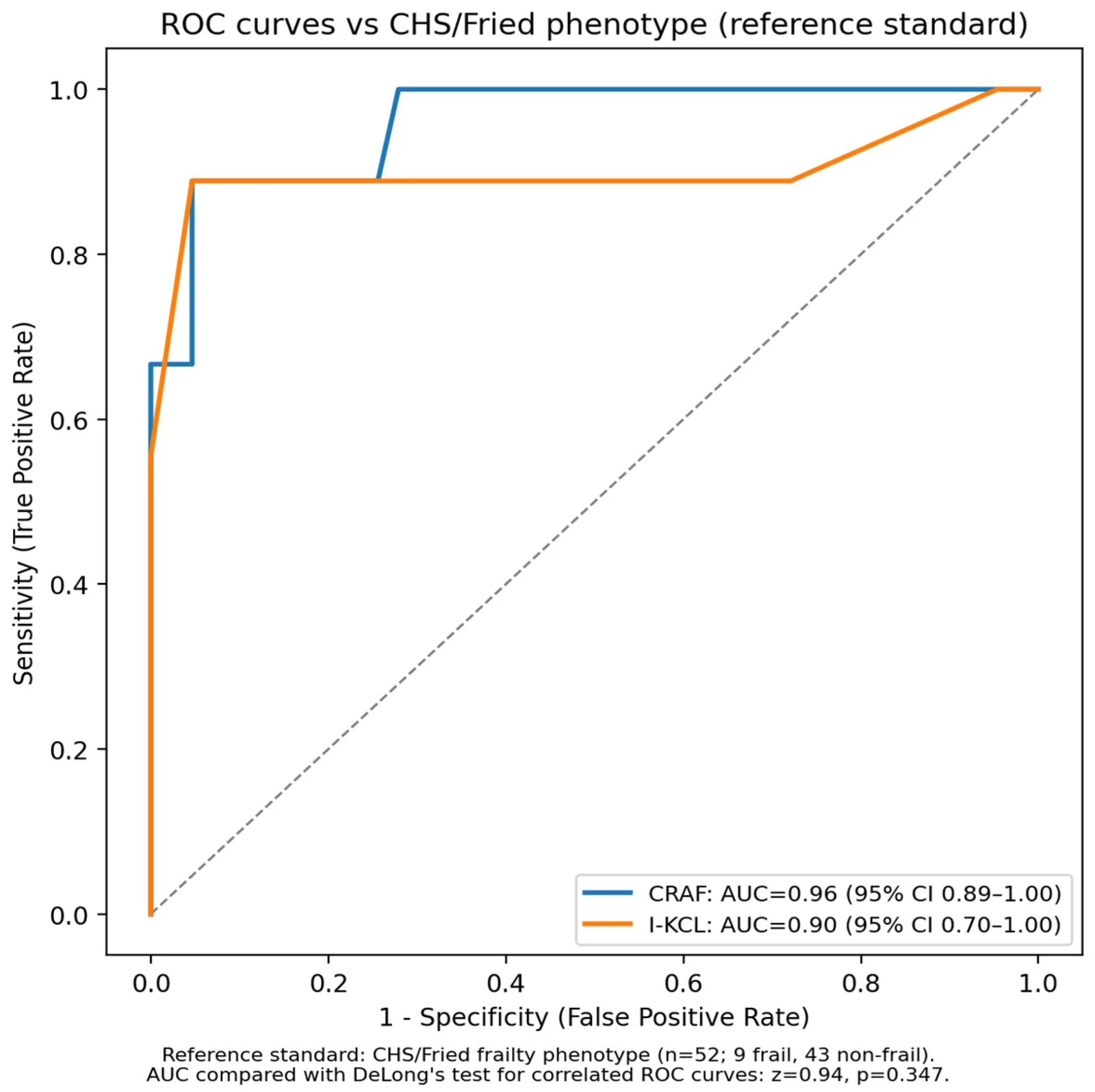
ROC curves for CRAF and I-KCL against the CHS/Fried phenotype reference standard, with AUC, 95% bootstrap confidence intervals, and DeLong test result for the AUC difference.

*ROC analysis, DeLong comparison, and sample size/power*. Discriminative accuracy of CRAF and I-KCL scores against the CHS/Fried phenotype reference standard was excellent for both instruments: CRAF AUC = 0.96 (95% CI 0.888–1, bootstrap, 2000 stratified resamples) and I-KCL AUC = 0.899 (95% CI 0.704–1) (Figure 4). Because the two ROC curves were generated from the same 52 patients against the same reference standard, DeLong’s test for correlated ROC curves was used to compare the AUCs (rather than the original, independent-sample Hanley and McNeil approach): z = 0.941, *p* = 0.3467. The difference between CRAF and I-KCL discriminative accuracy was therefore not statistically significant, supporting a complementary rather than hierarchical (superior/inferior) relationship between the two instruments. Bootstrap resampling (2000 replicates, stratified by CHS/Fried frailty status to preserve the observed 9:43 case-to-control ratio in every resample) was used to estimate the 95% confidence intervals for the AUCs because it does not rely on the asymptotic normality assumptions underlying closed-form variance estimators (e.g., the Hanley–McNeil formula), which can be unstable when the number of events is small, as in our frail subgroup (n = 9); 2000 replicates exceed the minimum of 1000–2000 that is generally recommended to obtain stable percentile-based confidence intervals for AUC statistics. Sample size and power were calculated using the Hanley–McNeil closed-form variance estimator for a single AUC tested against the null value of 0.5, applied to our observed case-mix (9 frail, 43 non-frail; frailty prevalence 17.3%). At the observed effect sizes, post hoc power to detect each AUC as significantly different from 0.5 (two-sided α = 0.05) exceeded 99% for both CRAF (AUC = 0.96, SE = 0.0466, power = 1) and I-KCL (AUC = 0.899, SE = 0.0711, power = 0.9999). Conversely, solving for the minimum total sample size required to achieve 80% power to detect these effect sizes at the same case-mix ratio yielded N = 10 (2 frail/8 non-frail) for CRAF and N = 15 (3 frail/12 non-frail) for I-KCL. Both figures are well below the 52 patients actually enrolled, confirming that the study was adequately and conservatively powered for its primary discriminative-accuracy objective. This calculation, with its full parameters (reference AUC, prevalence, α, target power), has been added to the Statistical Analysis Section in place of the previous unsupported statement that the sample size “provided more than 80% power.”

## 4. Discussion

Frailty is increasingly recognised as a clinically relevant construct in patients with PMR, reflecting a state of reduced physiological reserve and heightened vulnerability to internal and external stressors [5,6]. Although frailty has traditionally been conceptualised as a consequence of chronological ageing, accumulating evidence indicates that, in inflammatory rheumatic diseases, it is also strongly shaped by disease-related factors, including persistent systemic inflammation and cumulative comorbidity burden.

In our cohort, the prevalence of frailty defined by the CHS/Fried phenotype—the reference standard applied in this study—was 17.3% (9/52), closely comparable to the 17% reported by Sattui et al. using the same phenotype in a PMR cohort [11]. Using the CRAF and I-KCL instruments themselves, 19.2% of patients (10/52) were classified as frail by each tool, with corresponding pre-frailty rates of 44.2% (23/52) for CRAF and 36.5% (19/52) for I-KCL. These figures are numerically higher than, but of broadly similar order of magnitude to, those obtained with the CHS phenotype alone—an expected finding given that CRAF and I-KCL incorporate additional comorbidity, functional, and psychosocial domains beyond the five physical criteria of the Fried phenotype. Because CRAF, I-KCL, and the CHS phenotype rest on different conceptual models—deficit accumulation, multidimensional screening, and physical phenotype, respectively—and because the Sattui cohort differs from ours in setting and population, these prevalence figures should be interpreted as broadly concordant rather than as evidence of superior case-finding by any one instrument [11]. For accuracy, we also clarify that the sarcopenia (assessed by DXA), pain-behavior, and cognitive findings associated with frailty status in the published literature were reported by Sattui et al. in their own cohort [11]; these variables were not collected in the present study and are cited here as external supporting evidence for the clinical correlates of frailty in PMR, not as results of the present analysis. A plausible biological substrate linking chronic inflammation to frailty is “inflammaging,” a persistent, low-grade pro-inflammatory state associated with immunosenescence and cumulative cellular stress [8]. Elevated circulating interleukin-6 (IL-6) and tumour necrosis factor-α (TNF-α) have been consistently associated with frailty and pre-frailty in meta-analytic evidence from older adults, through mechanisms including catabolic effects on skeletal muscle, impaired muscle regeneration, and dysregulation of the hypothalamic–pituitary axis. PMR is, by definition, a systemic inflammatory disease characterised by persistently elevated IL-6 and CRP; the present findings—in which PMR-AS(CRP), a validated composite disease-activity index incorporating CRP, together with comorbidity count [41], emerged as the strongest independent correlates of both CRAF and I-KCL scores—are consistent with this inflammaging framework and support the hypothesis that disease-related inflammatory burden, rather than chronological age alone, drives much of the frailty signal observed in PMR.

In multivariable linear regression models adjusted for age, disease duration, PMR-AS(CRP), comorbidity count, HAQ, and fatigue, PMR-AS(CRP) (standardized β = 0.51, *p* < 0.001) and comorbidity count (standardized β = 0.36, *p* < 0.001) were independently associated with the CRAF score (adjusted R^2^ = 0.787, n = 51), while PMR-AS(CRP) (standardized β = 0.40, *p* < 0.001) and comorbidity count (standardized β = 0.46, *p* < 0.001) were independently associated with the I-KCL score (adjusted R^2^ = 0.675, n = 51). Age was not independently associated with either instrument (CRAF: B = 0.002/year, *p* = 0.333; I-KCL: B = 0.041/year, *p* = 0.289), nor were disease duration or fatigue. Variance inflation factors did not exceed 2.04 for any predictor in either model, and residuals did not depart significantly from normality (Shapiro–Wilk W = 0.977, *p* = 0.426 for CRAF; W = 0.988, *p* = 0.882 for I-KCL), indicating that multicollinearity and violation of regression assumptions are unlikely to explain these results. The absence of an independent association between age and either frailty score, despite age being the strongest correlate of frailty in general-population studies, deserves specific comment. We propose three, non-mutually exclusive explanations. First, PMR occurs almost exclusively after age 50, and the resulting restriction of age range in our cohort—compared with general-population cohorts spanning several decades—likely reduced the statistical variance available for age to demonstrate an independent effect. Second, age-related variance in frailty may be substantially mediated through disease-related factors already included in the model, particularly inflammatory burden and comorbidity count, effectively absorbing the marginal contribution of chronological age once these mediators are accounted for. Third, in a systemic inflammatory disease, disease-driven mechanisms such as cytokine-mediated sarcopenia and glucocorticoid myopathy may become dominant drivers of frailty, superseding chronological age as the principal correlate—an interpretation consistent with the broader literature on frailty in inflammatory rheumatic disease, where disease activity and inflammatory burden, rather than age alone, often emerge as the principal correlates of frailty [10,12,13]. A further finding was that functional impairment, measured by the HAQ, was independently associated with the CRAF score (standardized β = 0.19, *p* = 0.018) but not with the I-KCL score (standardized β = 0.06, *p* = 0.512). This pattern suggests that CRAF integrates aspects of disability into its overall frailty construct to a greater extent than I-KCL, which instead appears to capture a broader, more generic geriatric frailty construct that is comparatively independent of PMR-specific disability. This distinction has clinical relevance, since frailty and disability are overlapping but partially independent constructs with different prognostic implications. This finding, however, requires an important methodological caveat. Because the HAQ was also used, as a pragmatic proxy, to operationalize the “low physical activity” criterion within the CHS/Fried-based reference standard from which CRAF itself partly derives its conceptual structure, there is a partial construct overlap—and a plausible incorporation bias—between the HAQ and both the reference standard and CRAF. The magnitude of the CRAF–HAQ association reported here should therefore be interpreted with caution and cannot be assumed to be free of this circularity. Future validation studies should use an independent, activity-specific measure (e.g., the Minnesota Leisure Time Activity Questionnaire or accelerometry) rather than the HAQ, to disentangle genuine construct associations from artefactual overlap introduced by shared instrumentation. Despite these differences in construct associations, CRAF and I-KCL showed substantial categorical agreement: the linear-weighted Cohen’s kappa was 0.807 (unweighted κ = 0.76), corresponding to “almost perfect” agreement according to standard benchmarks, and Bowker’s test of symmetry did not detect a significant asymmetric disagreement pattern between the two classifications (χ^2^ = 2.0, df = 1, *p* = 0.157) [42]. Continuous scores were highly correlated (Spearman ρ = 0.745, *p* < 0.001) and did not differ systematically after standardization (Wilcoxon signed-rank W = 655, *p* = 0.757). Taken together, these results indicate that, at the level of overall categorical classification, CRAF and I-KCL are largely concordant, even though they diverge in which a specific clinical variable—functional disability—independently predicts their respective scores. Against the CHS/Fried phenotype as reference standard, both instruments showed excellent discriminative accuracy: the area under the ROC curve (AUC) was 0.960 (95% CI 0.888–1.0) for CRAF and 0.899 (95% CI 0.704–1.0) for I-KCL. Although the point estimate for CRAF was numerically higher, the difference between the two correlated AUCs did not reach statistical significance on DeLong’s test (z = 0.941, *p* = 0.347) [39]. We therefore revise our original interpretation: rather than concluding that CRAF has superior discriminative ability, our data indicate that CRAF and I-KCL achieve statistically comparable discriminative accuracy against the CHS phenotype in this PMR cohort, with CRAF additionally capturing PMR-specific functional impairment through its association with the HAQ, subject to the circularity caveat noted above. CRAF and I-KCL should accordingly be regarded as complementary, rather than hierarchically superior or inferior, instruments for frailty assessment in PMR.

Several methodological limitations warrant comment. First, the original CRAF mild- and moderate-frailty categories were collapsed into a single “pre-frailty” category for the purposes of this analysis, in order to align the three-category structure of CRAF with that used for I-KCL and the CHS phenotype and thereby enable direct cross-tool comparison. This recategorization was a study-specific analytic decision that has not itself been externally validated and should be considered when interpreting or replicating our frailty and pre-frailty prevalence estimates. Second, the cross-sectional design precludes causal inference regarding the relationships among frailty, disease activity, and comorbidity burden; longitudinal follow-up is needed to determine whether changes in inflammatory activity or treatment influence frailty trajectories over time. Third, although the achieved sample of 52 patients provided more than 80% post hoc power (Hanley–McNeil method) to detect AUC values as high as those observed here relative to the null value of 0.5, the ratio of observations to predictors in the multivariable regression models (approximately 8–9 observations per predictor, for six candidate predictors) remained below the conventionally recommended threshold of 10–15 observations per predictor; regression findings should therefore be regarded as hypothesis-generating pending replication in larger, adequately powered cohorts. Fourth, all patients were receiving low-dose oral glucocorticoid therapy, a plausible contributor to muscle atrophy, weakness, and functional decline. Current prednisone-equivalent dose was not entered as a covariate in the primary models presented here, and residual confounding by both current and cumulative glucocorticoid exposure, as well as by glucocorticoid-related comorbidities such as osteoporosis and myopathy, cannot be excluded; this represents an important direction for future analysis. Finally, no universally accepted gold standard for frailty exists: the CHS/Fried phenotype, deficit-accumulation indices, and multidimensional screening checklists rest on different conceptual models and may identify different patient subsets, and the use of a dichotomous CHS-based reference standard may oversimplify the multidimensional, graded nature of frailty and could contribute to misclassification of intermediate (pre-frail) states.

These findings support a nuanced, complementary approach to frailty assessment in PMR rather than the adoption of a single preferred instrument. Because I-KCL is a brief, self-administered checklist that does not require physical performance testing or laboratory data [19], it may be well suited to rapid screening in community, primary-care, or high-volume outpatient settings to flag patients who warrant more detailed rheumatological assessment. CRAF, which incorporates disease activity, comorbidity, and functional items and requires clinical/laboratory data typically available at a rheumatology visit [18], may be better suited to comprehensive, in-clinic evaluation aimed at guiding individualised functional-outcome monitoring and treatment planning once a patient has been flagged as at risk. We propose this sequential, two-step pathway—I-KCL for screening, CRAF for confirmatory/comprehensive assessment—as a hypothesis arising from the present cross-sectional comparison, to be prospectively validated in future studies.

In conclusion, in this cohort of patients with PMR, CRAF and I-KCL showed substantial categorical agreement, excellent and statistically comparable discriminative accuracy against the CHS/Fried phenotype, and convergent associations with disease activity and comorbidity burden, consistent with an inflammaging-driven model of frailty in this population. The two instruments diverged specifically in their association with functional disability, which was independently captured by CRAF but not by I-KCL, subject to the methodological caveat regarding shared HAQ-based instrumentation discussed above. Age, disease duration, and fatigue were not independently associated with either score, suggesting that frailty in PMR is more closely linked to inflammatory and comorbidity burden than to chronological age per se. Rather than establishing the superiority of either tool, these findings support a complementary role for CRAF and I-KCL in rheumatology practice—I-KCL as a low-burden screening instrument and CRAF as a more comprehensive, disease-specific assessment tool—to be confirmed in larger, longitudinal, multicenter studies.

## Figures and Tables

**Table 1:** Frailty status in PMR is reported according to CRAF, I-KCL, and CHS criteria. Categorical variables are expressed as number and percentage.

Parameters of PMR Patients	No Frail	Pre-Frail	Frail
Frailty status according to CRAF (%)	23 (44.2%)	19 (36.5%)	10 (19.2%)
Frailty status according to I-KCL (%)	19 (36.5%)	21 (40.4%)	12 (23.1%)
Frailty status according to CHS (%)	21 (40.4%)	18 (34.6%)	13 (25.0%)

Abbreviations: CRAF: Comprehensive Rheumatologic Assessment of Frailty; I-KCL: Italian version of Kihon Checklist; CHS: Cardiovascular Health Study; PMR: polymyalgia rheumatica.

**Table 2:** (**a**) Multivariable linear regression predicting CRAF score. B = unstandardized regression coefficient; SE = standard error; Std. β = standardized regression coefficient; VIF = variance inflation factor. (**b**) Multivariable linear regression predicting I-KCL score. B = unstandardized regression coefficient; SE = standard error; Std. β = standardized regression coefficient; VIF = variance inflation factor.

(**a**) CRAF score						
**Predictor**	**B (unstd.)**	**SE**	**95% CI**	**Std. β**	**VIF**	***p***
Age (years)	0.0021	0.0022	−0.0023 to 0.0065	0.075	1.39	0.3329
Disease duration (months)	−0.0046	0.005	−0.0147 to 0.0056	−0.062	1.1	0.3697
PMR-AS (CRP-based)	0.0237	0.004	0.0157 to 0.0317	0.51	1.72	<0.001
No. comorbidities	0.0434	0.0114	0.0204 to 0.0663	0.355	2.04	<0.001
HAQ score	0.0867	0.0351	0.0159 to 0.1575	0.192	1.42	0.0175
Fatigue VAS	0.0053	0.0068	−0.0083 to 0.019	0.055	1.14	0.4365
Constant	−0.7003	0.1583	—	—	—	<0.001
(**b**) I-KCL score						
**Predictor**	**B (unstd.)**	**SE**	**95% CI**	**Std. β**	**VIF**	***p***
Age (years)	0.0409	0.0381	−0.0358 to 0.1177	0.102	1.39	0.2885
Disease duration (months)	0.0786	0.088	−0.0986 to 0.2559	0.075	1.1	0.3762
PMR-AS (CRP-based)	0.2626	0.0698	0.1219 to 0.4033	0.398	1.72	<0.001
No. comorbidities	0.7895	0.1997	0.387 to 1.1919	0.455	2.04	<0.001
HAQ score	0.4071	0.6152	−0.8328 to 1.647	0.064	1.42	0.5116
Fatigue VAS	−0.1388	0.1186	−0.3778 to 0.1003	−0.101	1.14	0.2483
Constant	−6.2903	2.7732	—	—	—	0.0283

## Data Availability

The data are available if requested.

## Supplementary Materials

The following supporting information can be downloaded at: https://www.mdpi.com/article/10.3390/jcm15166403/s1, Table S1: the Italian version of Kihon Checklist (I-KLC) for frailty.
