# Supplementary material for: Frailty Assessment in Polymyalgia Rheumatica: Comparative Performance and Determinants of Clinical-Research Frailty Assessment (CRAF) and Kihon Checklist (KCL) Scores
Source: J Clin Med. 2026 Aug 19;15(16):6403. doi: 10.3390/jcm15166403 (PMC13513625; doi:10.3390/jcm15166403)
Supplement: Supplementary file 1 [file jcm-15-06403-s001.zip › jcm-4447452-supplementary.pdf]

Table S1: We provided the English version of the Kihon Checklist (I-KLC) for frailty items, translated in Italian language, keeping the original scoring cut-off system (0/1 values -no/yes-) for each functional, physical, nutritional, social, and mental health question

I-KCL Checklist Items translated in Italian version

**English language**	**Italian language**	**Cut off 0/1 (no/yes)**
	**Instrumental Activities of Daily Living & Social Functioning**	
Do you use public transportation by yourself?	*Viaggia con i mezzi pubblici da solo/a?*	
Do you manage your daily shopping by yourself?	*Provvede alle spese quotidiane da solo/a?*	
Do you manage your own savings/finances by yourself?	*Provvede ad amministrare i suoi risparmi da solo/a?*	
Do you visit your friends from time to time?	*Fa' visita ai suoi amici ogni tanto?*	
Do you turn to family or friends for advice?	*Si rivolge alla famiglia o agli amici per suggerimenti?*	
	**Physical Strength & Mobility**	
Do you usually go up stairs without the help of a handrail or wall?	*Sale le scale solitamente in assenza dell'aiuto del corrimano o del muro?*	
Do you usually get up from a chair without needing help?	*Si alza dalla sedia solitamente senza bisogno di aiuto?*	
Can you usually walk for 15 minutes straight?	*Riesce a camminare solitamente per 15 minuti di fila?*	
Have you fallen in the past year?	*E' mai caduto/a nell'ultimo anno?*	
Do you have a fear of falling when walking?	*Ha paura di cadere quando cammina?*	
	**Nutrition & Oral Function**	
Have you lost 2 kg or more of weight in the past six months?	*Ha perso due kg o più di peso negli ultimi sei mesi?*	
Body mass index (BMI) less than 18.5 kg/m^2, if yes, mark as positive.	*Indice di massa corporea in kg/m2, se meno di 18.5 segnare risposta positiva.*	
Compared to six months ago, do you have difficulty eating solid foods?	*Rispetto a sei mesi fa ha difficoltà nel mangiare cibi solidi?*	
Have you choked on a drink or soup recently?	*Le è andata di traverso una bevanda o una minestra recentemente?*	
Do you often experience a dry mouth?	*Le capita spesso di avvertire secchezza in bocca?*	

Social & Cognitive Function

Do you go out of the house at least once a week?	*Esce di casa almeno una volta a settimana?*
Do you go out less frequently compared to last year?	*Esce di casa meno frequentemente rispetto all'anno scorso?*
Do family or friends tell you that you have memory loss? (for example, telling you "you always ask the same things")	*La famiglia o gli amici le dicono che ha perso la memoria? (le dicono ad esempio"chiedi sempre le stesse cose")*
When making a phone call, do you need to look up the phone number?	*Quando effettua una chiamata ha la necessità di cercare il numero di telefono?*
Do you happen to not remember today's date?	*Le capita di non ricordare la data del giorno?*

Mental Health (Depressive Mood)

In the past two weeks, have you felt a lack of purpose in your daily life?	*Nelle ultime due settimane ha percepito una mancanza di scopo nella sua vita quotidiana?*
In the past two weeks, have you felt a lack of happiness in doing things you usually enjoyed?	*Nelle ultime due settimane ha percepito una mancanza di felicità nel fare ciò che solitamente le piaceva?*
In the past two weeks, have you felt difficulty doing things you used to do easily?	*Nelle ultime due settimane ha percepito difficoltà nel fare ciò che prima realizzava facilmente?*
In the past two weeks, have you felt useless and incapable?	*Nelle ultime due settimane si è sentito/a inutile e incapace?*
In the past two weeks, have you felt tired for no reason?	*Nelle ultime due settimane si è sentito/a stanco senza motivo?*
